# Exploring Health Professionals’ Knowledge and Perceptions of Telehealth Implementation in Multisite Public Hospitals of East Wollega Zone, Oromia, West Ethiopia: Cross-Sectional Study

**DOI:** 10.2196/71870

**Published:** 2025-12-04

**Authors:** Adugna Oluma, Tesfaye Abera Gudeta, Misganu Diriba Olana, Aliyi Benti Daba, Demiso Geneti Dinagde, Ashenafi Tesfaye Yadesa, Lammi Atomsa, Takele Mitiku Tesema

**Affiliations:** 1 Department of Nursing, School of Nursing & Midwifery, Institute of Health Sciences, Wollega University, Nek’emtē, Oromia, 395, Ethiopia, 251 0917814582

**Keywords:** East Wollega Zone, health professionals, knowledge, perception, telehealth

## Abstract

**Background:**

A significant gap exists among health professionals regarding telehealth due to limited knowledge and varying perceptions. This disparity is particularly pronounced in low-income regions, where numerous barriers hinder its implementation.

**Objective:**

This study aimed to assess the knowledge and perception of telehealth among health professionals in public hospitals in East Wollega, Oromia Region, Ethiopia.

**Methods:**

A study was conducted with 397 health professionals in public health facilities in East Wollega Zone, selected through systematic random sampling. Data were collected via self-administered questionnaires from March 2024 to June 2024 and entered into EpiData (version 3.1; EpiData Association) and analyzed using SPSS (version 24; IBM Corp). Descriptive statistics, including percentages and frequency distributions, were used. The Hosmer and Lemeshow test assessed variable homogeneity. Significant variables *(P<*.05) in bivariable logistic regression analysis were included in multivariable logistic analysis and reported as adjusted odds ratios (AOR) with 95% CI.

**Results:**

Findings indicated high levels of knowledge and perception among participants. Factors significantly associated with knowledge included educational status (AOR 4.22, 95% CI 3.45-13.12; *P*=.001), income between Ethiopian Birr 7001‐9000 (a currency exchange rate of Ethiopian Birr 52=US $1 during the study period; AOR 3.255, 95% CI 1.790-8.878; *P*=.001), access to computers (AOR 2.414, 95% CI 1.046-14.764; *P*=.001), consultation sessions (AOR 2.389, 95% CI 1.961-10.158; *P*=.001), and smartphone use (AOR 3.027, 95% CI 2.797-14.729; *P*=.001). For perception, significant associations included income over 9000 Birr (AOR 2.675, 95% CI 2.271-19.277; *P*=.001), improving quality of life (AOR 1.786, 95% CI 1.575-22.587; *P*=.009), increased accessibility (AOR 1.244, 95% CI 1.061-11.333; *P*=.04), consultation sessions (AOR 4.777, 95% CI 2.318-15.062; *P*=.001), and smartphone use (AOR 3.836, 95% CI 2.900-13.573; *P*=.001).

**Conclusions:**

Approximately two-thirds of participants demonstrated good knowledge and perception of telehealth. It is highly recommended to create awareness through educational programs for individuals with lower educational backgrounds and to improve access to technological devices.

## Introduction

In recent years, there has been significant interest in integrating telehealth into health care systems in both high- and low-income countries. Telehealth refers to the practice of providing medical care at a distance, using information and communication technologies to exchange valid information for diagnosis, treatment, disease prevention, research, and ongoing education of health care providers [1].

Telehealth strives to meet the needs of today’s health care consumers and transform health care delivery. It enhances the quality of care by improving accessibility and efficiency, reducing the need for travel, providing clinical support, and overcoming geographic barriers, ultimately leading to improved patient outcomes [2]. In high-income countries, telehealth can significantly benefit the health care sector through three main approaches: (1) real-time communication, which connects patients with health care providers via video conferencing, home health monitoring devices, and phone consultations; (2) store-and-forward, which allows for sharing images, data, voice, or video with physicians for long-distance diagnosis; and (3) remote patient monitoring, which involves tracking patients’ health parameters from home for ongoing evaluation and response [3].

Improving access to health services is crucial for achieving sustained improvements in health status. However, access to health care varies significantly between countries, particularly in low- and middle-income countries, which have less access to health services. Despite accounting for 90% of the world’s diseases, low- and middle-income countries receive only 12% of global health expenditures due to challenges associated with access, equity, quality, and cost-effectiveness [4]. Modern information and communication technologies are transforming how individuals communicate and seek information, addressing contemporary global health issues [5]. For instance, research in India found that a lack of understanding, skills, and appropriate working conditions hindered the implementation of electronic health systems, with about 12% of health professionals showing low awareness of telehealth [6]. Conversely, a study in Saudi Arabia indicated that 88.5% of health care professionals were interested in telemedicine technologies [5].

In Nigeria, approximately 60.9% of health professionals were familiar with telehealth, and 78.1% expressed a willingness to adopt it in health care delivery. Despite some external challenges, such as institutional organization and clinical practice guidelines, there is considerable acceptance of telehealth among health care professionals [7]. In the United States, a rising willingness to use telemedicine was noted, increasing from 57% in 2015 to 69% in 2019, particularly among younger physicians [8]. Telehealth effectively addresses geographic barriers, connecting patients with specialized providers more efficiently [8-10].

Health care management and telehealth services lie at the intersection of information technology, health care, and information science, aiming to address personnel shortages and enhance medical services through the use of information in biomedicine [11,12]. However, the Pan American Health Organization identified several barriers to telehealth implementation, including inadequate technological infrastructure, organizational challenges, and a lack of economic resources and scientific evidence regarding its benefits [13]. In Iraq, factors influencing telehealth adoption include privacy concerns, cultural attitudes, and the availability of technical support, with regulatory issues, lack of organizational support, and insufficient knowledge being major barriers [14,15].

In Ethiopia, the government has initiated a national e-health strategy to streamline various electronic health initiatives despite the challenges that remain, particularly in rural areas, due to population growth, economic constraints, and a shortage of medical specialists [16]. Even though a framework for adopting telehealth was initiated, the knowledge and perceptions of health care providers were not explored particularly in the study area. Therefore, this study aimed to identify knowledge gaps and perceptions toward telehealth among health care providers and their associated factors as well as to inform strategies for effective implementation, ensuring that the health care services are accessible and of high quality.

## Methods

### Study Design

An institutional-based cross-sectional study design was used.

### Study Setting and Population

The study was conducted in all 5 public hospitals in the East Wollega Zone of western Ethiopia, specifically 2 referral hospitals and 3 primary hospitals, from March 2024 to June 2024. The selected hospitals were Wollega Comprehensive Specialized Hospital, Nekemte Specialized Hospital, Gida Hospital, Arjo Hospital, and Sire Hospital. These public health facilities provide preventive, primary, general, referral, and specialized services, staffed by a total of 2917 health care providers. They serve a population of 1,500,999, comprising 726,741 females and 774,258 males, in both urban and rural areas of East Wollega Zone.

### Study Population and Samples

The study included all 5 public hospitals in East Wollega Zone. The sampling frame consisted of a list of health care professionals working in these hospitals. Based on the nature of service delivery, health care professionals included were physicians, medical laboratory technologists, anesthetists and anesthesiologists, pharmacists, nurses, and midwives. Informed consent was obtained from all selected participants.

### Eligibility Criteria

#### Inclusion Criteria

All health care professionals presented during the study period were included.

#### Exclusion Criteria

Health care professionals who were not willing to participate and not present during the study period were excluded.

#### Sample Size Determination

The sample size for the study was calculated using the formula for estimating a single population proportion, assuming a 95% CL and a marginal error (d) of 0.05. Since no previous studies in Ethiopia have addressed the variables proposed for this study, a population proportion of 50% will be considered.

Thus, the sample size will be


$$N=\frac{\left(z1-\alpha /2\right)2\times p\left(1-p\right)}{d^{2}}$$

$$ni=\frac{{\left(1.96\right)}^{2}*0.5\left(1-0.50\right)}{{\left(0.05\right)}^{2}}=384$$

By using a 10% nonresponse rate, the final sample size was 422.

### Sampling Procedures

For sampling and sample size calculation, the sampling frame was obtained from the respective human resources administrative offices, which provided the total number of health care providers working in the selected public hospitals of the East Wollega Zone; this was calculated to be 916. The total sample was calculated using proportional allocation and systematic random sampling techniques across all 5 hospitals.

To achieve proportional allocation, the constant K value was calculated, resulting in a value of 2. An initial number was randomly selected from 1 to 2, and a study participant was chosen at every second interval from all selected public health facilities in the East Wollega Zone. A total sample size of 422 was calculated from each hospital: Nekemte Specialized Hospital (n=159, 37.7%), Wollega University Comprehensive Specialized Hospital (n=122, 28.9 %), Sire Hospital (n=41, 10%), Arjo Hospital (n=47, 11%), and Gida Hospital (n=53, 13%)

### Data Collection Tools and Procedures

In this study, the data collection used a comprehensive 3-part questionnaire designed to gather relevant information from participants. The first part of the questionnaire focused on demographic information, where participants provided details about their personal and professional backgrounds. This section included questions regarding their names, ages, sex, designations, computer knowledge, and professions, allowing for a thorough understanding of the respondents.

The second part aimed to assess the knowledge level of the participants regarding telehealth, validated and adapted from a previous study [17]. It comprised 11 statements, each requiring a “1=Yes” or “0=No” response. This scoring system enabled participants to achieve a minimum score of 0 and a maximum score of 11, reflecting their knowledge about telehealth. Although we used surveys with identical items from those published in the literature, formal psychometric validation of this measure was not conducted. Therefore, observed differences should be interpreted as descriptive rather than indicative of true differences in the underlying of this construct.

The third part evaluated the respondents’ perceptions of telemedicine through 11 statements, adopted from previously validated tools [18]. Participants responded using a 5-point Likert scale, ranging from 0 (strongly disagree) to 4 (strongly agree). This section allowed for a cumulative score between 0 and 44, providing insights into how health care professionals perceived telehealth. Although we used surveys with identical items from those published in the literature, formal psychometric validation of this measure was not conducted. Therefore, observed differences should be interpreted as descriptive rather than indicative of true differences in the underlying of this construct.

To facilitate the data collection process, trained data collectors distributed the structured questionnaires to participants. A team of 4 data collectors and 2 supervisors was recruited to carry out this task over a period of 2 consecutive months.

### Study Variables

#### Dependent Variable

The dependent variables were knowledge and perception of telehealth.

#### Independent Variables

The independent variables include sociodemographic factors such as age, sex, education level, experience, and income. In addition, the study examined exposure to information communication technology, as well as the clinical applications and benefits of telehealth. This comprehensive approach allowed for a thorough analysis of the factors influencing knowledge and perception regarding telehealth.

### Operational Definition

#### Telehealth

Telehealth refers to the use of technology-driven virtual platforms to provide a range of health services, including information dissemination, disease prevention, monitoring, and medical treatment [19].

#### Knowledge

Knowledge refers to an individual’s understanding and awareness of the concepts, practices, technologies, and applications associated with telemedicine. It is measured using 11 questions, with responses coded as 1=“Yes” or 0=“No.” The minimum possible score is 0 and the maximum score is 11 [17]. Knowledge is divided into two types as follows: good knowledge where individuals score above the average mean on all knowledge-measuring questions, and poor knowledge where individuals score below the average mean on all knowledge-measuring questions.

#### Perceptions

The process of recognizing and interpreting telemedicine, measured on a 5-point Likert scale ranging from 0 (strongly disagree) to 4 (strongly agree), with a minimum score of 0 and a maximum score of 44 [18]. Perception is divided into two types as follows: good perception where respondents score above the average mean (>22) on all perception-measuring questions, and poor perception where respondents score below the average mean on all perception-measuring questions.

### Ethical Considerations

Official approval to conduct this study was obtained from the Office of Research and Technology Transfer Vice President at Wollega University. A letter of permission was then distributed to the respective hospitals, ensuring that all necessary approvals were in place to proceed with the study. Informed consent was obtained from all study participants, making it clear that their participation was voluntary and that they could withdraw at any time. The privacy of the study participants was ensured by removing personal identities, and their data were not disclosed to other parties. Moreover, participants' confidentiality was upheld by protecting their data, which was only shared for the purpose of the study based on the consent obtained.

### Data Quality Control

To ensure the quality of the data collected, all questionnaires were developed in English and subsequently translated into Afan Oromo, the local language. This translation was then retranslated back into English by experts to verify accuracy. A pretest was conducted with 5% of the sample size at Ghimbi General Hospital prior to the main data collection. Data collectors and supervisors received 1 day of training to prepare them for their roles. During the data collection phase, the data were cleaned, coded, and checked for consistency and completeness. A double-entry method was used to enhance data quality, allowing for cross-checking of any inconsistent entries and responses.

### Data Processing and Analysis

After data collection, the information was cleaned, edited, coded, and entered into EpiData (version 3.1; EpiData Association). It was then exported to SPSS Windows version 24 (IBM Corp) for analysis. Descriptive statistics, including percentages, ratios, frequency distributions, and pie charts, were used to summarize the data. The Hosmer and Lemeshow test will be used to evaluate the homogeneity of the variables. All variables that are significant at a *P*<.05 in the bivariable analysis were included in the multivariate logistic regression analysis. To ascertain the most suitable variables for the multivariate logistic regression, a backward stepwise goodness-of-fit approach was used. Finally, multivariate logistic regression analysis was conducted, reporting adjusted odds ratios (AORs) at 95% CIs, with a significance level set at *P*<.05.

### Dissemination of Results

The final report of the study was submitted in both soft and hard copies to the Wollega University Institute of Health Sciences Research Affairs Directorate, School of Nursing and Midwifery, Department of Nursing. The document was also distributed to each hospital participating in the study. In addition, the findings were presented in national and international workshops and conferences.

## Results

### Distribution of Sociodemographic Characteristics of the Participants

A total of 397 participants were enrolled in the study, resulting in a response rate of 94.1%. Among the respondents, the majority were male participants (n=238, 59.9%). Approximately half of the participants, 199 out of 397 (50.1%), were aged between 30 and 39 years. In terms of ethnic background, 382 out of 397 (96%) participants identified as Oromo, and Afan Oromo was the predominant language spoken by 383 out of 397 (96.5%) participants.

Regarding professional affiliation, most participants were nurses, comprising 221 out of 397 (55.7%), followed by physicians, who accounted for 60 out of 397 (15.1%). In addition, over half of the respondents, 227 out of 397 (57.2%), identified as Protestant. In relation to work experience, nearly half, 197 out of 397 (49.6%), reported serving for a duration of 6 to 10 years, with the majority earning a monthly wage between Ethiopian Birr 7001 and 9000 (a currency exchange rate of Ethiopian Birr 52=US $1 during the study period; Table 1).

**Table 1.** Distribution of sociodemographic characteristics of health professionals working in public hospitals of western Ethiopia, 2021 (N=397).

Variable	Value, n (%)
Sex	
Male	238 (59.9)
Female	159 (40.1)
Age (y)	
20‐29	143 (36.0)
30‐39	199 (50.1)
>40	55 (13.9)
Ethnicity	
Amhara	11 (2.8)
Oromo	382 (96.2)
Others^a^	4(1.0)
Religion	
Protestant	227 (57.2)
Orthodox	111 (28.0)
Muslim	52 (13.1)
Others	7 (1.8)
Marital status	
Single	161 (40.6)
Married	236 (59.4)
Language	
Afan Oromo	383 (96.5)
Amharic	9 (2.3)
Others^b^	5 (1.3)
Profession	
Nurse	221 (55.7)
Midwifery	53 (13.4)
Pharmacist	35 (8.8)
Medical laboratory technician	23 (5.8)
Medical doctor	60 (15.1)
Anesthetist and anesthesiologists	5 (1.3)
Experience (y)	
1‐5	77 (19.4)
6‐10	197 (49.6)
11‐15	85 (21.4)
>16	38 (9.6)
Income (Birr)^c^	
3000‐5000	30 (7.6)
5001‐7000	121 (30.5)
7001‐9000	183 (46.1)
>9000	63 (15.9)

a Others: Tigre and Guraghe.

b Others: Tigrigna and Guraghign.

c A currency exchange rate of Ethiopian Birr 52=US $1 during the study period.

### Health Professionals’ Exposure to Information and Communication Technology (ICT)

Participants’ familiarity with various types of information technology services is essential for the effective implementation of telehealth among health professionals. Regarding smartphone use, the majority, 246 out of 397 (62%), reported owning a smartphone. However, more than half, 220 out of 397 (55.2%), had never participated in training on computer systems and ICT, while over a third, 150 out of 397 (37.8%), had received introductory-level training. In addition, a significant portion, 305 out of 397 (76.8%), did not use computers regularly. Among those who did, 137 out of 397 (34.5%) primarily used computers for word processing and database management, while 113 out of 397 (28.5%) used them for entertainment purposes.

In terms of internet use, 167 out of 397 (42.1%) accessed the internet daily, followed by 72 out of 397 (18.1%) who reported weekly use. When examining the types of internet applications, more than half, 212 out of 397 (53.4%), indicated they used social media, followed by email and messaging services, which were used by 79 out of 397 (19.9%). Furthermore, regarding the use of medical-related websites, nearly two-thirds, 260 out of 397 (65.5%), reported that they had not visited such sites (Table 2).

**Table 2.** Exposure to information communication technology among health professionals working in public hospitals in East Wollega, western Ethiopia, 2021 (N=397).

Variables		Value, n (%)
Smartphone use		
Yes		246 (62)
No		151 (38)
Computer and ICT^a^ training		
Never attended		220 (55.4)
Introductory level		150 (37.8)
Certificate and above		27 (6.8)
Computer use		
Yes		92 (23.2)
No		305 (76.8)
Benefits of computer use		
Word processing and database management		137 (34.5)
Internet access		110 (27.7)
Entertainment		113 (28.5)
For more than 1 service		37 (9.3)
Frequency of internet use		
Every day		167 (42.1)
Twice a week		65 (16.4)
Every week		72 (18.1)
Every month		51 (12.8)
Less once a month		42 (10.6)
Types of internet application use		
Email services		79 (19.9)
Social media		212 (53.4)
Messaging applications		79 (19.9)
Cloud storage services		27 (6.8)
Medical-related website visits		
Yes		137 (34.5)
No		260 (65.5)

a ICT: information and communication technology.

### Clinical Applications and Benefits of Telehealth

In the context of increasing digitalization and technological advancements in health care, telehealth presents a range of clinical applications. This study revealed that the majority of telehealth applications—160 out of 397 (40.3%) participants—are focused on chronic conditions, followed by gynecological diseases, which accounted for 93 out of 397 (23.4%) participants. When considering the specific purposes of telehealth, nearly two-thirds, or 119 out of 397 (30%) participants, viewed it as a means to enhance accessibility and convenience, while one-fourth, 100 out of 397 (25.2%), believed it improves patients’ quality of life.

Regarding sources of information about telehealth, nearly two-thirds of participants reported that they learned about it from the internet (141 out of 397, 35.5%) and from colleagues (134 out of 397, 33.8%). Furthermore, more than two-thirds of the study participants, totaling 267 out of 397 (67.3%), had not attended telemedicine consultation sessions (Table 3).

**Table 3.** Clinical applications and benefits of telehealth among health professionals working in public hospitals in East Wollega, western Ethiopia, 2021 (N=397).

Variables		Value, n (%)
Clinical application of telehealth		
Gynecological disorder		93 (23.4)
Chronic diseases		160 (40.3)
Infectious diseases		38 (9.6)
Pediatrics disorders		25 (6.3)
Mental illness		46 (11.6)
For all		24 (6.0)
Do not know		11 (2.8)
Benefit of telehealth		
Improve quality of life		100 (25.2)
Improve access and convenience		119 (30)
Reduce cost		48 (12.1)
Reduce isolation		88 (22.2)
Ensure data safety and security		42 (10.6)
Telehealth information source		
Colleague		134 (33.8)
Internet		141 (35.5)
Workshop or training session		87 (21.9)
News and radio		17 (4.3)
Others^a^		18 (4.5)
Alternative tasks for difficult cases		
Refer to another hospital		265 (66.8)
Schedule an appointment		69 (17.4)
Review previous cases and literature		63 (15.9)
History of attendance in telehealth consultation sessions		
Yes		130 (32.7)
No		267 (67.3)

a Others: webinars, books, journals, and peer networking.

### Level of Knowledge and Perceptions of Implementing Telemedicine Among Health Professionals

The findings of this study revealed that 270 (68.01%) health professionals had a good level of knowledge about telemedicine, while 127 (32%) were poorly knowledgeable. Regarding perceptions of telehealth medicine implementation, 247 (62%) professionals expressed a positive perception, whereas 150 (38%) had a negative view.

### Bivariable Logistic Regression Analysis of Factors Associated With Knowledge of Telehealth Implementation

In the bivariable logistic regression analysis, several variables were found to be significantly associated with knowledge of implementing telehealth. Specifically, the factors identified include sex, educational level, work experience, computer and ICT training, level of income, computer use, previous attendance at telemedicine consultations, and smartphone use; all with a significance level of *P*<.05 (Table 4). These findings suggest that demographic and training-related factors may play a crucial role in enhancing health care professionals’ understanding and implementation of telehealth practices.

**Table 4.** Bivariable logistic regression analysis of factors associated with good knowledge of implementing telehealth among health professionals working in public hospitals of western Ethiopia, western Ethiopia, 2021 (N=397).

Variables		Knowledge^b^, n (%)		COR^a^ (95% CI)	*P* value
		Good	Poor		
Sex					
Male		141 (59.2)	97 (40.8)	2.958 (1.841-4.752)	.001^c^
Female		129 (81.1)	30 (19.9)	1	
Education					
Diploma		11 (40.7)	16 (59.3)	1	
Bachelor’s degree		177(66)	91 (34)	1.040 (1.261-6.348)	.012^c^
Master’s degree		32 (76.2)	10 (23.8)	1.538 (1.636-13.244)	.004^c^
Medical doctor		50 (83.3)	10 (16.7)	1.984 (2.610-12.266)	.001^c^
Experience (y)					
1‐5		34 (44.2)	43 (55.8)	1	
6‐10		136 (69)	61 (31)	0.322 (0.140-0.741)	.008^c^
11‐15		73 (85.9)	12 (14.1)	0.928 (0.423-1.949)	.805
>16		27 (71.1)	11 (28.9)	2.478 (0.978-6.279)	.056
Computer and ICT^d^ training					
Never attended		142 (64.5)	78 (35.5)	1	
Introductory level		104 (69.3)	46 (30.7)	0.106 (0.081- 0.986)	.047^c^
Certificate and above		24 (88.9)	3 (11.1)	0.096 (0.066-0.780)	.018^c^
Income (Birr)^e^					
3000‐5000		11 (36.7)	19 (63.3)	1	
5001‐7000		71 (58.7)	50 (41.3)	0.387 (0.278-1.667)	.395
7001‐9000		159 (86.9)	24 (13.1)	4.11 (1.23-9.12)	.001^c^
>9000		29 (46)	34 (54)	1.510 (0.910-3.075)	.103
Computer use					
Yes		157 (89.2)	19 (10.8)	2.067 (1.583-7.610)	.001^c^
No		113 (51.1)	108 (48.9)	1	
Attendance of telehealth consultation					
Yes		120 (92.3)	10 (7.7)	4.236 (2.700-18.641)	.001^c^
No		150 (56.2)	117 (43.8)	1	
Smartphone use					
Yes		136 (55.3)	110 (44.7)	4.852 (3.628-11.203)	.001^c^
No		134 (88.7)	17 (11.3)	1	

a COR: crude odds ratio.

b Dependent variable: knowledge.

c Significance at *P<*.05.

d ICT: information and communication technology.

e A currency exchange rate of Ethiopian Birr 52=US $1 during the study period.

### Multivariable Logistic Regression Analysis of Factors Associated With Knowledge of Implementing Telehealth

All variables significant at a *P<*.05 in the bivariable logistic regression were included in the multivariable logistic regression analysis. The following factors were found to be significantly associated with knowledge of implementing telehealth: higher educational level (Master’s degree and above), higher income level, access to computer usage, attendance at telehealth consultation sessions, and access to smartphone use.

Individuals with a higher educational status, such as MD and above, were nearly 3 times more likely to have knowledge of implementing telehealth compared to those with only a diploma (AOR 2.797, 95% CI 2.019-26.872). Those holding an MD and above were about as likely to be knowledgeable about implementing telehealth compared to diploma holders (AOR 4.22, 95% CI 3.45-13.12). Regarding income level, individuals earning between 7000 and 9000 birr monthly were more than 4 times more likely to be knowledgeable about implementing telehealth compared to those earning between 3000 and 5000 birr (AOR 4.11, 95% CI 1.23-9.12).

In terms of computer use, individuals with experience using computers were over twice as likely to be knowledgeable about implementing telehealth compared to those without such experience (AOR 2.414, 95% CI 1.046-14.764). Moreover, those who had attended telehealth consultation sessions were more than twice as likely to possess knowledge about implementing telemedicine compared to those who had not attended such sessions (AOR 2.389, 95% CI 1.961-10.158). Finally, smartphone users were nearly 3 times more likely to be knowledgeable about implementing telehealth compared to nonusers (AOR 3.027, 95% CI 2.797-14.729; Table 5).

**Table 5.** Multivariable logistic regression analysis of factors associated with knowledge of implementing telehealth among study participants, western Ethiopia (2021; N=397).

Variables		Knowledge^c^, n (%)		COR^a^ (95% CI)	*P* value	AOR^b^ (95% CI)	*P* value
		Good	Poor				
Education							
Diploma		11 (40.7)	16 (59.3)	1		1	
Bachelor’s degree		177 (66)	91 (34)	1.040 (1.261-6.348)	.012	1.539 (0.587-5.008)	.324
Master’s degree		32 (76.2)	10 (23.8)	1.538 (1.636-13.244)	.004	1.852 (1.689-14.044)	.006
MD and above		50 (83.3)	10 (16.7)	1.984 (2.610-12.266)	.001	4.22 (3.45-13.12)	.001^d^
Income (Birr)							
3000‐5000		11 (36.7)	19 (63.3)	1		1	
5001‐7000		71 (58.7)	50 (41.3)	0.387 (0.278-1.667)	.395	1.558 (0.696-32.410)	.112
7001‐9000		159 (86.9)	24 (13.1)	2.050 (4.033-14.960)	.001	3.255 (1.790-8.878)	.001^d^
>9000		29 (46)	34 (54)	0.510 (0.910-3.075)	.103	1.291 (0.949-13.940)	.060
Computer use							
Yes		157 (89.2)	19 (10.8)	2.067 (4.583-13.610)	.001	2.414 (1.046-14.764)	.001^d^
No		113 (51.1)	108 (48.9)	1		1	
Attendance of telehealth consultation							
Yes		120 92.3)	10 (7.7)	2.236 (4.700-18.641)	.001	2.389 (1.961-10.158)	.001^d^
No		150 (56.2)	117 (43.8)	1		1	
Smartphone use							
Yes		136 (55.3)	110 (44.7)	1.852 (3.628-11.203)	.001	3.027 (2.797-14.729)	.001^d^
No		134 (88.7)	17 (11.3)	1		1	

a COR: crude odds ratio.

b AOR: adjusted odds ratio.

c Dependent variable: knowledge of implementing telehealth.

d Significance at *P *value ≤.05.

In the bivariable logistic regression analysis, several variables were found to be significantly associated with the perception of implementing telehealth. Specifically, the factors identified include sex, work experience, computer and ICT training, level of income, computer usage, previous attendance at telehealth consultations, daily internet use, perceived benefits of telemedicine for improving quality of life, accessibility of services, convenience, and smartphone use at a significance level of *P*<.05 (Table 6).

**Table 6.** Bivariable logistic regression analysis of factors associated with perceptions of implementing telehealth among health professionals working in public hospitals of western Ethiopia (N=397).

Variables		Perception^a^, n (%)		COR^b^ (95% CI)	*P* value^c^
		Good	Poor		
Sex					
Male		106 (45.5)	132 (55.5)	0. 476 (10.310-0.733)	.001
Female		44 (27.7)	115 (72.3)	1	
Experience (y)					
1‐5		12 (15.6)	65 (84.4)	0.185 (0.076-0.447)	.001
6‐10		79 (40.1)	118 (59.9)	0.669 (0.334-1.344)	.259
11‐15		19 (50)	19 (50)	0.889 (0.413-1.911)	.763
>16				1	
Computer and ICT^d^ training					
Never attended		121 (55)	99 (45)	0.549 (0.071-0.635)	.006
Introductory level		103 (68.7)	47 (31.3)	0.965 (0.125-1.164)	.090
Certificate and above		23 (85.2)	4 (14.8)	1	
Income (Birr)^e^					
3000‐5000		7 (23.3)	23 (76.7)	1	
5001‐7000		33 (27.3)	88 (72.7)	1.157 (0.412-3.322)	3.32
7001‐9000		97 (53)	86 (47)	0.866 (0.695-2.992)	.325
>9000		13 (20.6)	50 (79.4)	3.467 (2.207-8.526)	.001
Computer use					
Yes		91 (51.7)	85 (48.3)	1.807 (1.482-3.390)	.001
No		156 (70.6)	65 (29.4)	1	
Attendance of telehealth consultation					
Yes		82 (63.1)	48 (36.9)	3.609 (3.187-7.842)	.001
No		68 (25.5)	199 (74.5)	1	
Smartphone use					
Yes		186 (75.6)	60 (24.4)	3.520 (2.957-7.075)	.001
No		61 (40.1)	90 (59.6)	1	
Frequency of internet use					
Every day		114 (87.7)	16 (12.3)	4.781 (1.623-21.722)	.007
Twice a week		34 (47.2)	38 (52.8)	1.294 (0.209-2.665)	.652
Every week		18 (35.3)	33 (64.7)	0.788 (0.122-1.699)	.241
Every month		37 (39.8)	56 (60.2)	0.376 (0.196-2.408)	.557
Less once a month		33 (82.5)	7 (17.5)	1	
Not at all		9 (81.8%)	2 (18.2%)	1.368 (0.931, 16.582)	.063
Benefit of telehealth					
Improve quality of life		15 (15)	85 (85)	4.159 (3.811-19.709)	.001
Improve access and convenience		35 (29.7)	83 (70.3)	2.288 (1.752-7.510)	.001
Reduce cost		34 (70.8)	14 (29.2)	0.462 (0.263-1.507)	.299
Reduce isolation		40 (45.5)	48 (54.5)	1.607 (0.874-3.852)	.108
Ensure data safety and security		26 (60.5)	17 (39.5)	1	

a Dependent variable: perceptions of implementing telehealth.

b COR: crude odds ratio.

c Significance at *P*≤.05.

d ICT: information communication technology.

e A currency exchange rate of Ethiopian Birr 52=US $1 during the study period.

### Multivariable Logistic Regression Analysis of Factors Associated With Perceptions of Implementing Telehealth

All variables that were significant at a *P* value of <.05 in the bivariable logistic regression were included in the multivariable logistic regression analysis. The following factors were found to be significantly associated with perceptions of implementing telehealth: higher income level, work experience, attendance at telehealth consultation sessions, perceived benefits of telehealth, and access to smartphone use.

Individuals with 1 to 5 years of experience were less likely to have a positive perception of implementing telehealth compared to those with more than 16 years of experience (AOR 0.0562, 95% CI 0.020-0.233). Regarding income level, individuals earning more than 9000 birr monthly were nearly 3 times more likely to have a positive perception of implementing telehealth compared to those earning between 3000 and 5000 birr (AOR 2.675, 95% CI 2.271-19.277).

In terms of the perceived benefits of telemedicine, individuals who experienced its use for improving quality of life were nearly twice as likely to have a positive perception of implementing telehealth compared to those who perceived it primarily for ensuring data safety and security (AOR 1.786, 95% CI 1.575-22.587). In addition, individuals who perceived telehealth as a means of improving accessibility and convenience were 1.244 times more likely to have a positive perception compared to those focused on data safety and security (AOR 1.244, 95% CI 1.061-11.333).

Moreover, those who had attended telehealth consultation sessions were nearly twice as likely to possess a positive perception of implementing telehealth compared to those who had not attended such sessions (AOR 4.777, 95% CI 2.318-15.062). Finally, smartphone users were nearly 2 times more likely to have a positive perception of implementing telehealth compared to nonusers (AOR 3.836, 95% CI 2.900-13.573)(Table 7).

**Table 7.** Multivariable logistic regression analysis of factors associated with perceptions of implementing telehealth among health professionals working in public hospitals of western Ethiopia (2021; N=397).

Variables		Perception^c^, n (%)		COR^a^ (95%)	*P* value	AOR^b^	*P* value
		Good	Poor				
Experience (y)							
1‐5		12 (15.6)	65 (84.4)	0.289 (0.076-0.447)	.001	0.056 (0.020-0.233)	.001^d^
6‐10		79 (40.1)	118 (59.9)	0.401 (0.334-1.344)	.259	0.139 (0.024-1.263)	.051
11‐15		19 (50)	19 (50)	0.918 (0.413-1.911)	.763	1.095 (0.027-2.556)	.066
>16				1		1	
Income (Birr)							
3000‐5000		7 (23.3)	23 (76.7)	1		1	
5001‐7000		33 (27.3)	88 (72.7)	1.157 (0.412-3.322)	3.322	0.512(0.205-13.547)	.632
7001‐9000		97 (53)	86 (47)	1.366 (0.695-2.992)	.325	1.277(0.813-15.820)	.092
>9000		13 (20.6)	50 (79.4)	2.467 (2.207-8.526)	.001	2.675(2.271-19.277)	.001^d^
Attendance of telemedicine consultation							
Yes		82 (63.1)	48 (36.9)	4.609 (3.187-7.842)	.001	4.777(2.318-15.062)	.001^d^
No		68 (25.5)	199 (74.5)	1		1	
Smartphone use							
Yes		186 (75.6)	60 (24.4)	3.520 (2.957-7.075)	.001	3.836 (2.900-13.573)	.001^d^
No		61 (40.1)	90 (59.6)	1		1	
Benefit of telehealth							
Improve quality of life		15 (15)	85 (85)	4.159 (3.811-19.709)	.001	1.786 (1.575-22.587)	.009^d^
Improve access and convenience		35 (29.7)	83 (70.3)	2.288 (1.752-7.510)	.001	1.244 (1.061-11.333)	.040^d^
Reduce cost		34 (70.8)	14 (29.2)	0.462 (0.263-1.507)	.299	1.222 (0.163-3.942)	.801
Reduce isolation		40 (45.5)	48(54.5)	1.607(0.874, 3.852)	.108	2.817 (0.604-8.478)	.225
Ensure data safety and security		26 (60.5)	17(39.5)	1		1	

a COR: crude odds ratio

b AOR: adjusted odds ratio.

c Dependent variable = perceptions of implementing telehealth.

d Significance at *P*≤.05.

## Discussion

### Main Findings

The limited implementation of telehealth in Ethiopia, with less than 10% adoption, can be attributed to several factors hindering its advancement [20,21]. In this cross-sectional study, we aimed to assess the level of knowledge and perception of health professionals regarding telemedicine implementation in the East Wollega Zone of the Oromia region, western Ethiopia. We identified significant factors associated with the knowledge and perception of telehealth implementation among targeted individuals, which contribute to the digitalization of global health care systems for better access to health care services.

### Comparison With Prior Work

The results indicated that approximately 68.01% (270/397) of participants possessed a good level of knowledge, which exceeds that reported in previous research conducted in referral hospitals in northwest Ethiopia (56.4%, 37.6%, 65.8%, and 40.7%), and Saudi Arabia (46.1%) [18,22-25]. Conversely, the knowledge level in our study was higher than findings from Nigeria and Iran (34.5% and 3.9%, respectively) [26,27] . This disparity may be linked to lower accessibility to information and training regarding telehealth at the national level, geographical impact to access relevant resources, and impact of the COVID-19 pandemic, as studies conducted during the outbreak.

Furthermore, our findings revealed that 62% (247/397) of health professionals held a positive perception of implementing telehealth, surpassing the results of a study conducted in Addis Ababa, Ethiopia, where approximately 60.9% of professionals expressed a positive perception toward telehealth [28,29]. In contrast, it was lower compared to the studies reported in India, where 73.14% of participants had a positive perception, and in Saudi Arabia, where the figure was 90%; another study reported 65.2% positive perception of implementing telemedicine in India [18,29,30]. These differences may reflect variations in technological advancement, accessibility, and economic conditions across the regions studied.

In this study, higher educational status was identified as an independent factor associated with knowledge of telehealth, considering the joint effects of other variables. Professionals with a higher level of education were 3 times more likely to possess good knowledge of implementing telemedicine compared to those with a lower educational level, such as a diploma. This finding aligns with a study conducted in northern Ethiopia, which revealed that individuals with a degree or higher had significantly increased knowledge scores related to telehealth [31]. These similarities suggest that a higher educational level contributes to greater exposure to various sources of information about telehealth, thereby enhancing understanding.

Moreover, the findings of this study indicated that health professionals with access to smartphones were nearly twice as likely to have a positive perception of implementing telehealth compared to non-users. This is consistent with a study conducted in central Ethiopia, which found that participants with sufficient access to computers or smartphones were more likely to have a favorable perception of using telehealth than those lacking adequate devices (32). This similar finding underscores the importance of accessibility to technological resources for exchanging information and disseminating knowledge about the purpose of telehealth, thereby enhancing positive beliefs toward its implementation [28].

### Study Limitations

This study has several limitations as follows:

1. Self-reported data: the study relies on self-reported measures of knowledge and perceptions, which may introduce bias. Respondents might overestimate their knowledge or provide socially desirable answers.
2. Design approach: as a cross-sectional study, it captures data at a single point in time, which may not reflect changes in knowledge or perceptions over time or lacks causal-effect relationship.
3. Potential response bias: participants may have differing levels of familiarity with telehealth, leading to variations in responses that could affect the study’s outcome.
4. Limitation of psychometrics properties testing: although we used surveys with identical items from those published in the literature, formal psychometric validation of this measure was not conducted in this study.

### Conclusions

In conclusion, the findings of the study revealed a relatively higher level of knowledge and perception in implementing telehealth. Educational status, income, computer and smartphone use, and exposure to telehealth training were factors associated with the knowledge of implementing telehealth, whereas income level, work experience, exposure to telehealth training sessions, perceived benefits of telehealth, and access to smartphone use were factors associated with the perception of telemedicine.

Based on the study’s findings, it is recommended to enhance educational programs for health professionals to improve their knowledge of telehealth, particularly targeting those with lower educational backgrounds. Increasing access to smartphones and computers, along with providing regular training sessions and awareness campaigns about the benefits of telehealth, will further bolster positive perceptions. In addition, addressing income disparities and fostering collaborative learning environments can significantly contribute to a more favorable attitude toward telehealth implementation.

## Abbreviations

AOR: adjusted odds ratio
ICT: information and communication technology
